# Probing the mechanistic role of the long α-helix in subunit L of respiratory Complex I from *Escherichia coli* by site-directed mutagenesis

**DOI:** 10.1111/j.1365-2958.2011.07883.x

**Published:** 2011-11-07

**Authors:** Galina Belevich, Juho Knuuti, Michael I Verkhovsky, Mårten Wikström, Marina Verkhovskaya

**Affiliations:** Helsinki Bioenergetics Group, Institute of Biotechnology, PO Box 65 (Viikinkaari 1), FIN-00014, University of HelsinkiHelsinki, Finland

## Abstract

The C-terminus of the NuoL subunit of Complex I includes a long amphipathic α-helix positioned parallel to the membrane, which has been considered to function as a piston in the proton pumping machinery. Here, we have introduced three types of mutations into the *nuoL* gene to test the piston-like function. First, NuoL was truncated at its C- and N-termini, which resulted in low production of a fragile Complex I with negligible activity. Second, we mutated three partially conserved residues of the amphipathic α-helix: Asp and Lys residues and a Pro were substituted for acidic, basic or neutral residues. All these variants exhibited almost a wild-type phenotype. Third, several substitutions and insertions were made to reduce rigidity of the amphipathic α-helix, and/or to change its geometry. Most insertions/substitutions resulted in a normal growth phenotype, albeit often with reduced stability of Complex I. In contrast, insertion of six to seven amino acids at a site of the long α-helix between NuoL and M resulted in substantial loss of proton pumping efficiency. The implications of these results for the proton pumping mechanism of Complex I are discussed.

## Introduction

**Fig. 1:**
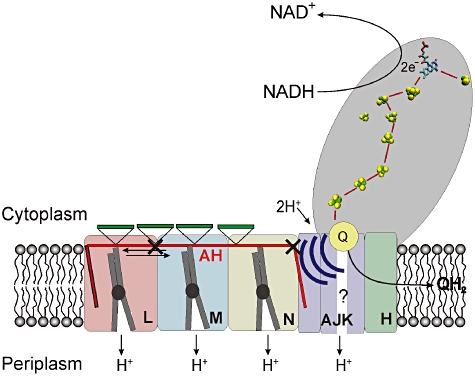
Possible interruption of AH role in ‘piston’ model of Complex I by mutations. The scheme of ‘piston’ model is based on (Efremov *et al*., 2010). The amphipathic α-helix (red) derived from NuoL and anchored with two (15th and 16th) TMHs at the distal and proximal ends of the membrane domain, respectively, is in tight contact with the discontinuous α-helices that presumably form H^+^ channels in all three antiporter-like subunits, NuoM, N and L. Reduction of ubiquinone initiates conformational changes (violet arcs) that push AH, which causes reorganization of the proton channels and synchronous release of 3 H^+^ at the periplasmic side of the membrane. The fourth proton is suggested to be pumped through a putative proton channel formed by the small subunits, NuoA, J and K. Truncation of the NuoL subunit in the C-terminus was performed at the sites indicated by crosses. The estimated locations of four insertion/substitution sites in the long α-helix are indicated by green bars.

NADH:ubiquinone oxidoreductase type 1 (Complex I) constitutes the beginning of the respiratory chain in the membranes of mitochondria and many bacteria. The redox reaction catalysed by Complex I is coupled to proton translocation across the membrane, which contributes to generation of protonmotive force and synthesis of ATP. The structural arrangement of Complex I differs fundamentally from other redox-linked generators of protonmotive force in that no redox centres are found in the membrane domain of the enzyme, which argues against direct coupling between electron transfer and proton translocation. Upon NADH oxidation, electrons are transported from the primary electron acceptor, flavine mononucleotide (FMN), to a chain of several FeS clusters arranged along the hydrophilic domain of Complex I that protrudes out of the membrane on the N-side (negatively charged side of the coupling membrane). The last FeS cluster (called N2) reduces ubiquinone that binds near the interface of the hydrophilic and membrane domains (for reviews see Sazanov, 2007; Ohnishi and Nakamaru-Ogiso, 2008; Zickermann *et al*., 2008). Proton pumping is driven by the redox reaction, but must be carried out by protein subunits in the membrane arm. Most notably, three large subunits, NuoL, NuoM and NuoN, located next to each other along the membrane arm (Fig. 1), share a pattern of conserved and functionally essential amino acid residues with certain cation/proton antiporters (Fearnley and Walker, 1992; Hiramatsu *et al*., 1998; Mathiesen and Hagerhall, 2003; Morino *et al*., 2010). Evidently, free energy from the redox reaction must be transferred across a long distance (∼ 100Å) to the distal membrane subunits, but until last year there was little information about how this might occur. The resolution of the structure of the membrane part of Complex I from *Escherichia coli* and *Thermus thermophilus* provided an interesting possibility. It was found that the most distal NuoL subunit in the membrane domain has a unique feature: the large fragment between the 15th and the last, 16th, transmembrane helices is organized as an amphipathic α-helix (AH) that runs along almost the entire length of the membrane domain on the N-side, and is anchored by the 16th TMH (transmembrane α-helix) in the proximal part of the domain (Fig. 1) (Efremov *et al*., 2010; Efremov and Sazanov, 2011). A similar but somewhat shorter ‘horizontal’α-helix was observed in the 3D structure of mitochondrial Complex I from *Yarrowia lipolytica* (Hunte *et al*., 2010). A ‘piston model’ (Fig. 1) was proposed to explain how the energy may be transferred over a long-distance in the membrane domain (Efremov *et al*., 2010; Ohnishi, 2010; Efremov and Sazanov, 2011). Delivery of a pair of electrons from NADH to ubiquinone was proposed to cause conformational changes in the proximal membrane subunits leading to movements of the long AH. Such movements of AH, which is in contact with transmembrane helices that may form proton-conducting channels in the L, M and N subunits, accomplish transmembrane proton translocation as envisaged schematically in Fig. 1. In this view, AH plays the role of a mechanical piston or a connecting rod that propagates long-distance conformational changes.

To test this concept, we generated multiple mutations in the NuoL subunit that may be expected to interrupt the function of AH as a piston. For this purpose we designed truncations of NuoL at the C- and N-termini, and a number of mutations within the long α-helix. The latter included replacements of the reasonably conserved residues Asp542, Lys551 and Pro552, and substitutions and insertions of six to seven amino acid residues in different positions of the helix. Here, we report the effect of these mutations on production and stability of Complex I, and on ubiquinone reductase activity and proton pumping efficiency. Overall, the data do not support a role of the long α-helix as a mechanical piston.

## Results

### Generation of mutations in the NuoL subunit

All obtained mutant strains are listed in Table 1. A first set of mutations included deletion of significant DNA fragments from *nuoL* resulting in a truncated NuoL or loss of the entire subunit. Truncation the NuoL at the N-terminus (see *Experimental procedures*) resulted in the loss of 370 amino acids following residue 5, therefore the first 11 transmembrane α-helices were lost. These segments share an amino acid pattern with corresponding fragments of NuoN, NuoM, and some multisubunit cation/proton antiporters, which suggests that they participate in H^+^ translocation. C-terminally truncated NuoL was devoid of the proximal half of the long α-helix, including the last (16th) TMH, or lacked only the 16th TMH as shown in Fig. 1. In the NuoL-deficient strain the *nuoL* gene was deleted from the chromosomal DNA and replaced by a Km^R^ cassette.

**Table 1:** Bacterial strains

Strains	Genotype/relevant properties	Reference
GR70N	F^-^*thi rpsL gal*, Sm^R^; wild-type Complex I	Green *et al*. (1984)
GRL3	GR70N, Δ*nuoL*::Km^R^; ΔNuoL, Km^R^, Sm^R^	This study
D542R, D542N K551Q, K551E P552A, P552C, P552Q	Single amino acid substitutions in NuoL	This study
GRLn	N-terminal truncation of NuoL; deletion of first 11 TMHs	This study
Y590St	C-terminal truncation of NuoL; deletion of the last 16th TMH	This study
K551fs	C-terminal truncation of NuoL; deletion of half an AH and the last 16th TMH	This study
As*ub^n^/ins^n^*	*sub/ins* indicates amino acid substitutions or insertions, after indicated amino acid A. *^n^*– number of amino acids in the sequence:	This study
	*n* = 6: DGDGDP	
	*n* = 7: PDGDGDP	

A second set of mutations consisted of single amino acid substitutions in the AH. The AH does not have a clear conserved pattern of amino acid sequence, and although it is rich in basic and acidic residues, none of them can be considered highly conserved. Hence, we chose the most conserved among other charged residues D542 and K551 to be replaced with an oppositely charged or a neutral residue. Moreover, the partially conserved P552 was substituted with Ala, Cys or Gln.

**Fig. 2:**
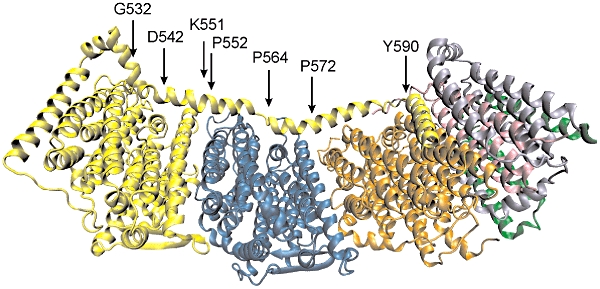
Estimated location of mutation sites in AH. A view of the membrane domain of *E. coli* Complex I (3RKO, PDB). Mutation sites in AH are indicated by arrows.

The intention with the last set of mutations was to decrease the rigidity and change the geometry of AH. For that purpose we introduced either a ‘soft’ insertion or a substitution with a number of amino acid residues, DGDGDP (*ins*^6^) or PDGDGDP (*ins*^7^/*sub*^7^), that predicted (PSIPRED) to form an unstructured flexible loop preserving the rest of the structure (Bryson *et al*., 2005). Such insertions/substitutions were positioned in the proximal, distal or middle part of the AH, as indicated by the green bars in Fig. 1. The location of all mutations was chosen on the basis of the crystal structure of the membrane domain of Complex I from *E. coli* (Efremov *et al*., 2010; Efremov and Sazanov, 2011). The estimated positions are close to possible contacts of AH with the antiporter-like membrane subunits, L, M and N, or they are located in the well-organized α-helical fragments of AH between L and M or M and N subunits as shown in Fig. 2. Table 2 lists the positions of the insertions/substitutions.

**Table 2:** Growth phenotype, Complex I activity and relative H^+^ pumping efficiency measured in membrane vesicles

		Activity of Complex I,^a^ %		
			
Mutation	Growth on minimal malate medium	dNADH:HAR oxidoreductase	dNADH:DQ oxidoreductase	Relative H^+^ pumping efficiency,^a^ %
Wt, GR70N^b^	+	100 ± 9	100 ± 12	100 ± 5
GRL3 (Δ*nuoL*)	−	7 ± 4	3 ± 3	nd
Truncation				
GRLn (*nuoL* N-truncated)	−	26 ± 1	11 ± 1	nd
Y590St (*nuoL* C-truncated)	−	42 ± 8	7 ± 3	nd
K551fs (*nuoL* C-truncated)	−	37 ± 2	14 ± 10	nd
Replacement of single amino acid residue				
P552A	+	100 ± 9	121 ± 7	109 ± 2
P552C	+	88 ± 7	108 ± 2	103 ± 3
P552Q	+	96 ± 5	120 ± 7	98 ± 4
D542R	+	61 ± 9	64 ± 12	102 ± 9
D542N	+	78 ± 8	91 ± 5	105 ± 6
K551Q	+	89 ± 14	104 ± 13	96 ± 6
K551E	+	80 ± 1	98 ± 3	96 ± 1
Insertion/substitution				
G532*ins*^7^	+	86 ± 5	84 ± 8	107 ± 15
G532*sub*^7^	+	94 ± 5	89 ± 12	103 ± 4
G532*ins*^6^	+	121 ± 12	117 ± 16	108 ± 7
P552*ins*^6^	−	65 ± 3	32 ± 2	22 ± 4
P552*ins*^7^	−	66 ± 11	25 ± 7	27 ± 2
P564*sub*^7^	+	42 ± 6	12 ± 5	nd
P564*ins*^6^	+	84 ± 9	78 ± 2	88 ± 1
P572*ins*^6^	+	52 ± 5	10 ± 3	nd

a Results are expressed in mean ± SD of two to four independent membrane preparations.

b One hundred per cent of dNADH:HAR reductase and dNADH:DQ reductase activities were 1.33 and 0.92 µmol dNADH mg^−1^ min^−1^ respectively.

### Growth phenotype, Complex I production and activity in membranes of generated mutants

The routine test of Complex I deficiency is the capability of *E. coli* cells to grow in the minimal medium containing malate as the sole energy source. The mutant strain with complete NuoL deletion, GRL3, as well as the three strains bearing the N- and C-terminally truncated NuoL, showed very slow growth in this medium (Fig. S1). In contrast, all variants with point mutations in the AH grew with rates comparable to wild type. The growth phenotype of the variants with the insertions/substitutions in the AH depended on the position of the mutation (Fig. S2). Only P552*ins*^6^ and P552*ins*^7^ were unable to grow in the test medium. Note especially that all three variants with substitution of P552 grew normally. The dNADH (reduced nicotinamide hypoxanthine dinucleotide, specific Complex I substrate):HAR [hexaammineruthenium (III) chloride] oxidoreductase activity in the bacterial membranes is often used as a reporter of Complex I production/assembly, although it is not always an accurate parameter (see below). Lack of this activity in membranes isolated from GRL3 (Table 2), taken together with the impaired growth of this strain in minimal malate medium indicates that no fully assembled Complex I was produced if *nuoL* was deleted from the chromosome. N-terminal truncation of NuoL led to a significantly reduced amount of Complex I; the membranes of the GRLn strain exhibited 26% of wild-type dNADH:HAR oxidoreductase and 11% of quinone reductase activity. The production of Complex I in the mutants bearing NuoL truncated at the C-terminus, Y590St and K551fs, was higher 42% and 37% of wt, respectively, but negligible quinone reductase activity was observed.

All mutants with replacement of a single amino acid residue exhibited an almost wild-type phenotype except for D542R that produced less Complex I, *c*. 60% of wild type. Still also membranes of D542R mutant gained full activity (both HAR and DQ reductase) when the cells were collected at the late exponential phase of growth (not shown). Substitution of seven amino acid residues and insertions, either *ins*^6^ or *ins*^7^ after G532, as well as *ins*^6^ after P564 in AH, also yielded normally produced and fully active Complex I. The phenotype of the variants with the substitution of seven amino acid residues after P564, and insertion into the position after P572, seemed contradictory at first glance because the cells grew well in malate medium (Fig. S2) although Complex I activity was extremely low in the isolated membranes. To solve this contradiction the activity was measured in spheroplasts. In this case DQ (decylubiquinone) reductase activity, which was fully rolliniastatin sensitive, was the same as in wild type (0.4 µmoles mg^−1^ min^−1^) in both variants, P572*ins*^6^ and P564*sub*^7^. After cell breakage to make membranes this activity dropped precipitously and became 10–12% of wild type (Table 2), which indirectly indicates that Complex I is normally produced and functional in these mutants, but highly unstable so that cell breakage causes severe enzyme damage.

The insertions into AH at residue P552, yielded strains that did not grow in malate medium, but which produced a significant amount of Complex I (their membranes possessed 65% HAR reductase activity) and exhibited reduced quinone reductase activity (32% and 25%, in P552*ins*^6^ and P552*ins*^7^; Table 2).

### Proton pumping efficiency of mutated Complex I

**Fig. 3:**
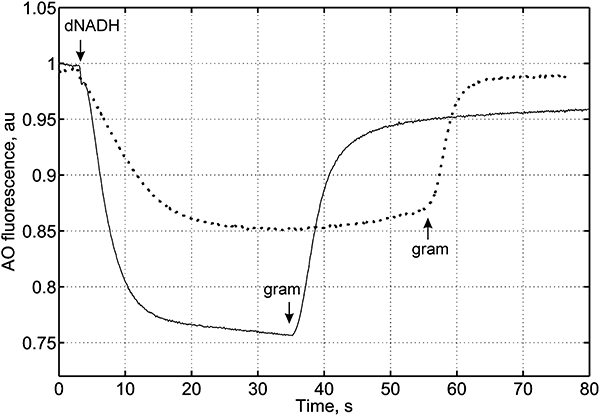
Proton translocation in *E. coli* membrane vesicles. The reaction was initiated by the addition of 80 µM dNADH; after approaching the stationary level, ΔpH was dissipated by the addition of gramicidin, 1 µg ml^−1^ what is indicated by arrows. Solid line: two pumps, Complex I and a terminal oxidase operate sequentially. Dotted line: Complex I operates alone: vesicles were pre-incubated with 5 mM KCN and the medium was supplemented with 100 µM DQ.

All variants, except those that lost most of the quinone reductase activity in the membranes (GRL3 GRLn, Y590St, K551fs, P564*sub*^7^ and P572*ins*^6^), were tested for efficiency of H^+^ pumping in inverted membrane vesicle preparations. Since the H^+^ translocation efficiency in the strains containing mutated Complex I is important for interpretation of the results, we carefully assessed the most widely used method, which is based on a comparison of the rates of dNADH oxidation and acidification of the interior of the membrane vesicles due to H^+^ translocation. This method does not yield numerical values of the H^+^ : e^-^ ratio but it is solid for obtaining the comparative values which is illustrated in Fig. 3. This figure shows H^+^ translocation in wild-type membrane vesicles initiated by dNADH addition and monitored by acridine orange (AO) fluorescence changes. The solid line represents H^+^ translocation by the sequential operation of two proton pumps in the respiratory chain of *E. coli*, viz. Complex I and ubiquinol oxidase (mainly *bo_3_* since the bacterial cells were grown at high aeration where *bd* oxidase is downregulated). The dotted line represents H^+^ translocation by Complex I alone, in which case the vesicles were incubated with KCN to block the terminal oxidase (no proton pumping by terminal oxidase was observed under these conditions), and the medium was supplemented with decylubiquinone (DQ) as the acceptor of reducing equivalents from dNADH. In both cases the generated ΔpH was dissipated by the addition of gramicidin. In the first case, the expected proton translocation stoichiometry is 4H^+^/e^-^[stoichiometry of 2H^+^/e^-^ in Complex I (Wikström, 1984; Galkin *et al*., 1999; Galkin *et al*., 2006) and 2H^+^/e^-^ in *bo_3_* oxidase (Puustinen *et al*., 1989; Puustinen *et al*., 1991)], whereas it is 2H^+^ per electron in the second case; i.e. the stoichiometric efficiency should differ by a factor of two. Since the electron transfer is in both cases limited by Complex I activity it is the same in both conditions. Thus the difference in H^+^ pumping in the two experiments shown in Fig. 3 should reflect the relative H^+^/e^-^ stoichiometry. Calculation of the relative proton pumping efficiency as a ratio between the rates of electron and proton transfer indeed showed the expected result: the efficiency is twice higher in case of two pumps. As can be seen in Fig. 3, both the initial slope and the steady-state level of H^+^ transfer differ in the two cases. In principle, both reflect the pumping efficiency at the same rate of electron flux. However, the steady-state level is determined by the balance between H^+^ pumping and H^+^ leakage in the opposite direction, of which the latter depends on the generated ΔpH and the proton permeability of the membrane. The permeability can vary not only between mutant strains, but also in different wild-type membrane preparations. For these reasons the steady-state level of acidification is not the best reporter of pumping efficiency. The rate of acidification depends linearly on the rate of electron transfer at the beginning of the reaction when the proton pump is working against zero ΔpH. Therefore, the ratio of these two parameters is a more dependable reflection of the pump stoichiometry.

**Fig. 4:**
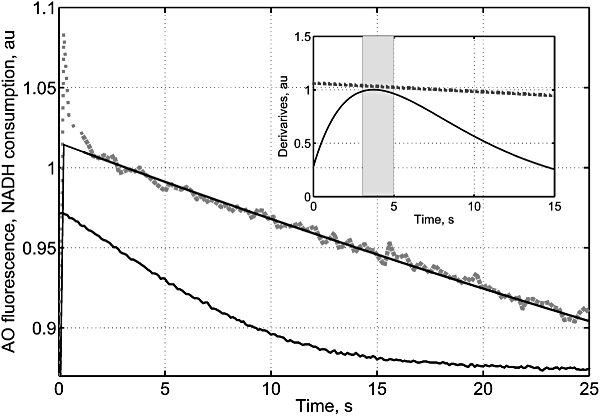
Determination of H^+^ pumping efficiency of Complex I. H^+^ translocation (solid line) and dNADH:ubiquinone reductase activity (dotted grey line) were monitored in the same conditions in the presence of 5 mM KCN and 100 µM DQ (main panel). The reaction was initiated by the addition of 80 µM dNADH at zero time. dNADH:ubiquinone reductase activity was fitted (solid line drawn through dotted grey line). The derivatives of the curves in the main panel representing the rates of e^-^ and H^+^ transfer are shown in the inset. The time window used for determination of the pumping efficiency is indicated by the grey bar.

The energy-dependent acidification of the interior of the membrane vesicles is relatively fast. To slow it down and hence to improve resolution of the initial rate, the vesicles were loaded with 200 mM buffer, pH 7.5 (see *Experimental procedures*: *Proton pumping activity measurements*). Figure 4 shows how the proton pumping efficiency was determined by the ratio between the proton and electron transfer rates. dNADH oxidation and H^+^ translocation were measured in the same conditions (main panel) and the derivatives of both curves were calculated (inset). The rate of electron transfer stays almost constant during the first 10–15 s, and then it gradually decreases due to the accumulation of reduced DQ (not shown). The rate of H^+^ translocation is more complicated. During the first 2–3 s it increases – due to mixing and activation of Complex I. Later on it decreases due to proton backflux that is accelerated by the growing ΔpH. Although the time window for determination of the efficiency is short (about 2 s; marked in grey in the inset), this approach is applicable. Figure 3 shows that the pumping efficiency of two protons pumps working sequentially, Complex I and *bo_3_* oxidase, is twice higher, as expected, than pumping efficiency when Complex I is working alone, which proves the reliability of this approach. However, at low electron transfer activity the H^+^ translocation rate could become comparable to the membrane leakage which would decrease the measured relative stoichiometry. To determine the range of Complex I ubiquinone reductase activity, where the approach is valid, we previously titrated Complex I with rolliniastatin and determined the pumping efficiency. We found that it was not dependent on the electron transfer rate in the range 15–100% of ubiquinone reductase activity in membranes (Euro *et al*., 2008). Due to this limit we were able to determine the pumping efficiency only in the mutants whose quinone reductase activity exceeded 15% of wild type.

**Fig. 5:**
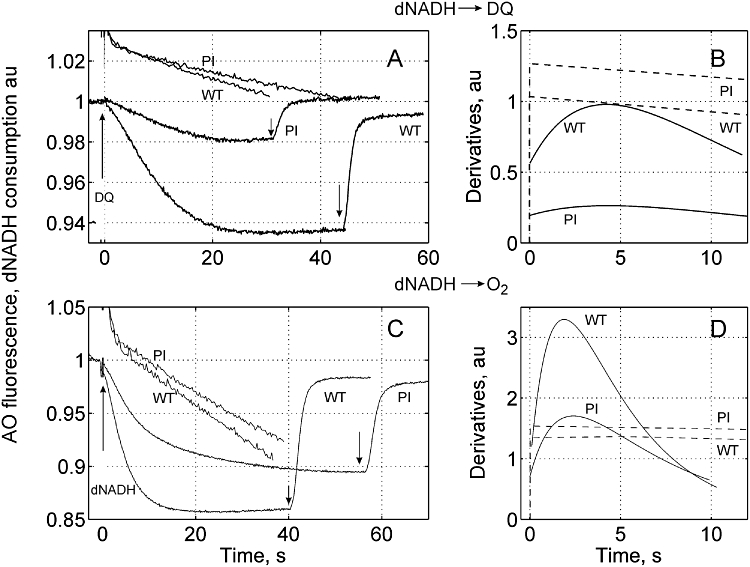
Impaired proton pumping in the P552*ins*^6^ Complex I. The acidification of the wild-type and P552*ins*^6^ membrane vesicle interior was initiated by the addition of 100 µM DQ (upper panel) or 80 µM dNADH (lower panel), and dissipated after approaching steady-state by addition of 1 µg ml^−1^ gramicidin A as indicated by arrows. Complex I from wild type was inhibited with 3 nM rolliniastatin to obtain a ubiquinone reductase activity close to that of P552*ins*^6^. Upper panel: acidification of the vesicle interior due to operation of Complex I alone. The vesicles were pre-incubated with 5 mM KCN, and the medium was supplemented with 80 µM dNADH. The reaction was initiated by the addition of 100 µM DQ, an artificial response on DQ addition is subtracted. Lower panel: acidification of the vesicle interior due to sequential operation of two proton pumps, terminal oxidase and Complex I. The reaction was initiated by the addition of 80 µM dNADH. Left panels (A, C): acidification of the vesicle interior and NADH consumption. Right panels (B, D): derivatives of the initial rates of proton (solid lines) and electron (dashed lines) transfer by wild type and P552*ins*^6^ (PI) Complex I.

All mutants that exhibited significant quinone reductase activity, except P552*ins*^6^ and P552*ins*^7^ (see Table 2), were found to pump protons with an efficiency varying from 85% to 110% of wild type (Fig. 4). In the P552*ins*^6^ and P552*ins*^7^ variants the rate of ubiquinone reduction was lowered to *c*. 25–30% of wild type, but also the H^+^/e^-^ ratio was decreased, down to 20–25% of wild type (cf. below). It is clearly seen by comparison of the proton pumping by wild-type Complex I inhibited with rolliniastatin to a rate close to that of the P552*ins* variants. Figure 5 shows the proton pumping by Complex I from P552*ins*^6^ alone (in the presence of KCN) (upper panel) and by Complex I and terminal oxidase (lower panel). In the first case the efficiency of the P552*ins*^6^ variant was roughly 20–25% of wild type. When Complex I and *bo_3_* oxidase operate in sequence in the absence of KCN and DQ, endogenous quinone mediates electron transfer between Complex I and the oxidase. In this case the stoichiometry of proton translocation in the P552*ins*^6^ vesicles was about 50% of that in wild type (Fig. 5, lower panel), which supports the contention that the efficiency of proton pumping in Complex I is severely compromised by the insertion.

No significant changes in membrane permeability were found in all tested mutants, since the pumping efficiency of the terminal oxidase (the reaction was initiated by the addition of dithiothreitol and ubiquinone-1) was identical (not shown).

### Purification of the mutated Complex I

Trials to purify Complex I with truncated L subunit, Y590st, were unsuccessful, because the enzyme proved to be unstable and was partially destroyed after the first steps of purification. Upon gel filtration peak exhibiting NADH:HAR oxidoreductase activity was shifted to lower-molecular-weight proteins indicating that it had lost the whole L subunit. The P552*ins*^6^ could be purified and the fraction that ran in the standard Complex I peak position on the gel filtration column contained all Complex I subunits (Fig. S3). The activity of the purified P552*ins*^6^ variant was 106 and 3.55 µmoles mg^−1^ min^−1^ for NADH:HAR and dNADH:quinone reductase respectively. The artificial HAR reductase activity of the mutant was only slightly lower than that in wild type (120–150 µmoles mg^−1^ min^−1^) whereas DQ reductase activity was only 12–18% of wild type (20–30 µmoles mg^−1^ min^−1^ in wt). The ratio of oxidoreductase activities (d)NADH:HAR and (d)NADH:DQ of the mutated Complex I was significantly raised upon purification: in membranes it was 3 (1.5 in wt), after isolation it was about 30, which means that the mutation makes the purified enzyme fragile and almost incapable of performing its natural catalytic action.

## Discussion

The role of the NuoL subunit in Complex I function was studied because of its probable role in transmembrane H^+^ translocation, and especially due to the unique amphipathic α-helix (AH) running along almost the entire length of the membrane domain (Efremov *et al*., 2010; Efremov and Sazanov, 2011). Results published so far on NuoL are contradictory. Steimle *et al*. (2011) reported that deletion of the *nuoL* gene from the *nuo* operon in *E. coli* resulted in close to wild-type electron transfer activity and twice lowered proton translocation efficiency, whereas Nakamaru-Ogiso *et al*. (2010) did not even detect assembly of Complex I in the Δ*nuoL* mutant. Our results are in agreement with the latter report. Lack of dNADH:HAR activity in the membranes from the Δ*nuoL* mutant clearly indicates loss of enzyme production or assembly. Judgement of assembly and production of intact Complex I was based on dNADH:HAR oxidoreductase activity throughout this study. We found that even truncation of NuoL at the C-terminus is highly deleterious; the Y590St and K551fs variants produced only *c*. 40% of Complex I with negligible quinone reductase activity, and hence they also lacked proton pumping activity. In contrast, the similar mutants, Y544St and W592St, studied by Steimle *et al*. (2011) were reported to have 80–85% of wild-type ubiquinone reductase activity and 50% proton pumping efficiency. We cannot explain this fundamental difference in results, but we note that the quinone reductase activity of the isolated wild-type enzyme reported by Steimle *et al*. was very low, approximately 14 turnovers per second, which is about 6% of that reported for this enzyme in other studies (Sazanov *et al*., 2003; Verkhovskaya *et al*., 2011).

Gradual loss of dNADH:HAR oxidoreductase activity of the Y590St mutant upon purification indicates fragility of Complex I when the NuoL subunit is truncated from the C-terminus. The truncation of 11 TMHs from the N-terminus yielded in only a quarter of the wild-type amount of the enzyme, as judged from the dNADH:HAR oxidoreductase activity, quinone reductase activity was practically completely lost. Here, the most conserved TMHs, viz. 5, 6, 7 and 8, were missing. These conserved helices share a sequence pattern with NuoM, NuoN and with multisubunit cation/proton antiporters, and are hence likely to be involved in proton translocation which is also supported by analysis of the resolved structure of the *E. coli* Complex I membrane domain (Efremov and Sazanov, 2011). It is important to note that although only one, the distal NuoL, of three similar fragments suggested to translocate H^+^ is lacking (see Fig. 1) the ubiquinone reductase activity was totally lost. This observation suggests that Complex I functions as an entity; i.e. the other proton-pumping subunits proximal to L cannot function either when vital parts of subunit L are lost. This notion is also supported by the observation that a number of point mutations in NuoL make the enzyme incapable to reduce ubiquinone (Nakamaru-Ogiso *et al*., 2010). Since Complex I function is fully reversible, the electron and proton transfers must be tightly coupled despite the fact that these two events are well separated both spatially and temporally. The electrons are transferred within the hydrophilic fragment and the protons are translocated in the membrane domain. The electron is delivered from NADH to cluster N2, the immediate donor to ubiquinone, in about 100 µs (Verkhovskaya *et al*., 2008), whereas the turnover time of Complex I is 2–3 ms. To minimize energy dissipation the enzyme should have a tight connection between the site of the electron and the distant elements of proton translocation, which means that the state of *each* proton translocating element should exhibit feedback to the ubiquinone binding site in order to allow further turnover after accomplished H^+^ transfer.

We undertook mutagenesis studies of the long α-helix (AH) in the C-terminus of NuoL that has been suggested to deliver the energy released upon ubiquinone reduction to the proton channels in the NuoN, M and L subunits by a mechanical motion, like a piston in a steam engine (Efremov *et al*., 2010; Ohnishi, 2010). To fulfil such a function AH should make tight contact with the proton channel elements in subunits L, M and N, and be rigid in order to transfer the energy with minimal losses. In our studies we addressed both these conditions. We performed a search for conserved amino acid residues in AH to find potential contact sites, but it did not show clearly conserved patterns. P552, which is located in the regular α-helical fragment of AH between NuoL and NuoM subunits I (3RKO, PDB), is partially conserved. Nevertheless, replacement of this proline with alanine, cysteine or glutamine yielded a wild-type phenotype (Table 2). The contact between AH and proton channels could be realized through electrostatic interactions between basic and acidic residues. Although AH is rich in charged residues in all organisms, none of them is fully conserved. We replaced the partially conserved D542 and K551 with neutral and oppositely charged residues, but no significant changes in electron or proton transfer activities were found, indicating that their side-chains are not directly involved in the pumping mechanism. The mutant D542R showed reduced (60–65%) Complex I production and DQ reductase activity, nevertheless its proton pumping efficiency was unchanged.

Several insertions and substitutions into the long α-helix were made with amino acid residues that hardly can form an α-helix, most of which yielded a wild-type phenotype or a somewhat fragile enzyme that fell apart on isolation of membrane vesicles, but showed wild-type growth at the cell level. Making the AH more flexible, or both more flexible and longer, at several positions in its distal and proximal parts had little or no effect on ubiquinone reductase activity or on proton pumping efficiency. These findings reinforce our impression that the detailed structure of the AH cannot be essential for the proton-pumping mechanism. In contrast to this, the mutants with insertions into AH at position after P552, between the L and M subunits (Fig. 2), did not grow in the malate medium. Nevertheless, the P552*ins*^6^ and P552*ins*^7^ variants were reasonably well produced (65%) and exhibited reduced albeit significant ubiquinone reductase activity (25–30%). However, their stoichiometric proton pumping efficiency was only one-fifth to one-fourth of wild type.

Overall, our results suggest that the AH is hardly a mechanical piston, but rather appears as a clamp in the Complex I structure that may be necessary to keep the subunits L, M and N together during catalytic turnover. The P552*ins*^6^ and P552*ins*^7^ variants are important because they are the examples of Complex I phenotypes where the coupling between electron transfer and proton translocation is strongly compromised. In mechanically coupled device as Complex I is thought to be, any conclusions on the mechanism should be made on the basis of the loss of the proton pumping efficiency. If AH is a coupling rod the insertion of the flexible loop between the L and M subunits is expected to lower pumping efficiency to three-fourths since only one antiporter-like subunit, NuoL, would be out of connection. In the P552*ins*^6^ and P552*ins*^7^ variants the pumping efficiency was decreased much more, to 20–25% of wild-type pumping which is not in good accord with the piston notion.

The insertion and substitution at the P564 position are located in the kink region of AH where there is a contact with the M subunit. These mutants yielded different phenotypes. The insertion resulted in slightly lower activities than wild type, but the enzyme with the substitution was rendered highly fragile, which could be explained by the importance of the residues following P564, L565 and N566, for the contact between AH and NuoM. According to the resolved structure (3RKO, PDB), these residues are closest to the NuoM subunit and they are deleted upon substitution but present after the insertion. It was surprising that the insertion at position P572 also had a drastic effect on Complex I stability although the structure does not show close contacts in this area. However, there is still a possibility that Complex I undergoes conformational changes upon turnover that affect the contacts between AH and NuoN.

## Experimental procedures

### Bacterial strains and site-directed mutagenesis

The mutants were obtained on the base of *E. coli* strain GR70N (Green *et al*., 1984). All genetic manipulations were carried out using JM109 (Promega) and XL10-Gold (Stratagene) *E. coli* strains. Mutagenesis of *nuoL* was carried out using QuikChange Lightning and QuikChange II XL Site-Directed Mutagenesis Kits (Stratagene) according to the manufacturer's instructions. Oligonucleotides (Eurofins MWG Operon) used for PCR and mutagenesis are listed in Table S1. The final plasmid with ready construct for gene replacement procedure and bearing PCR products as well as the template plasmid for mutagenesis were sequenced using an appropriate set of oligonucleotides to cover full length of *nuo* genes involved in the work. Mutations were confirmed by DNA sequencing of the template plasmid after mutagenesis and of the PCR product amplified from genomic DNA of the mutant strain.

### Generation of the NuoL-knockout strain and the template vector

The NuoL-deficient strain was constructed by deleting the *nuoL* gene from chromosomal DNA of the *E. coli* GR70N strain, and replacing it by the kanamycin resistance gene. For this purpose up- and downstream flanks of *nuoL* were amplified from genomic DNA using UpLF-UpLXhoR and DnLXhoF-DnLR oligonucleotide pairs and GoTaq DNA polymerase (Promega), and cloned separately into the pGEM-T Easy vector (Ap^R^, PCR product cloning vector, Promega), resulting in pGUpL and pGDnL respectively. Flanks were combined by ligating the downstream flank extracted by XhoI and SbfI digestion from pGDnL in the same restriction sites of pGUpL. The kanamycin resistance cassette extracted by SalI digestion from pUC4K (AC X06404) was inserted between flanks in the XhoI site yielding pGflL::Km^R^. The NgoMIV–SpeI fragment of pGflL::Km^R^ containing the kanamycin resistance cassette, with flanks complementary to the corresponding regions on chromosomal DNA, was cloned into pKO3 [pSC101-ts, *sacB*, *cat* (Cm^R^); gene replacement vector] digested by XmaI and XbaI. The resulting vector was used for transformation of GR70N competent cells and gene replacement as described by Link *et al*. (1997) yielding the NuoL-knockout strain GRL3. For generation of the template plasmid the *nuoL* gene together with flanking regions was amplified from genomic DNA of the *E. coli* GR70N strain using UpLF and DnLR oligonucleotides and AccuTaq LA DNA polymerase (Sigma-Aldrich). The *c*. 3.8 kb PCR product was cloned into the pGEM-T Easy vector and the resulting plasmid pG-ULD was used as the DNA template for site-directed mutagenesis.

### NuoL truncated at the N- or C-terminus

For N-terminally truncated NuoL *c*. 1 kb of the upstream flank of *nuoL*, including the short 5′ sequence of *nuoL* and 1.8 kb fragment containing the C-terminal part of *nuoL* encoding the NuoL subunit from position of Tyr376 and its downstream flank, were amplified by PCR using UpLF-UpLNheR and LnNheF-DnLR oligonucleotide pairs, respectively, and ligated separately into the pGEM-T Easy vector. Further, both parts were fused in pGEM in the NheI site generated due to oligonucleotide design. The final strain GRLn has deletion of a *c*. 1.1 kb segment of chromosomal DNA encoding the sequence of the 6th to the 375th amino acid of the NuoL subunit that is predicted to form the first 11 transmembrane helices.

For C-terminally truncated NuoL a new stop codon was introduced at the predicted beginning of the last (16th) TMH by mutagenesis of the template vector using sense Y590StF and antisense Y590StR oligonucleotides that finally resulted in the Y590St strain with deletion of 24 amino acids at C-terminus of NuoL. A second variant of C-terminally truncated NuoL was obtained accidentally: one of the mutagenesis reactions led to lack of one nucleotide in the K551 codon and a frameshift (fs) that resulted in a nucleotide sequence encoding nine new amino acids and a stop codon. This mutation is predicted to produce a NuoL subunit truncated in the middle of the long α-helix between the 15th and 16th transmembrane helices. The corresponding strain was named K551fs.

Restitution of the mutated *nuoL* gene on the chromosome was performed using the pKO3 vector, as described above, and the NuoL-knockout strain GRL3. Integration of the mutated *nuoL* gene into the chromosome by a double-cross-over event resulted in loss of kanamycin resistance.

### Bacterial growth and purification of Complex I

Bacteria were routinely grown in LB medium at 37°C with appropriate antibiotics at indicated concentrations (µg ml^−1^): ampicillin (100), streptomycin (50), kanamycin (50) and chloramphemicol (20). Growth tests were carried out under aerobic conditions in minimal malate medium (48 mM Na_2_HPO_4_, 22 mM KH_2_PO_4_, 0.05% NaCl, 18.7 mM NH_4_Cl, 1 mM MgSO_4_, 90 mM dl-malic acid, 0.1% yeast extract, pH 7.1). For Complex I purification cells were grown in LB medium at 37°C in a 25 l fermentor and harvested at the late exponential growth phase, the membranes were prepared by passing the cells through an APV Gaulin homogenizer as described in Belevich *et al*. (2007). Complex I was purified in three chromatography steps using anion exchanger DEAE-Trisacryl M (Bio-Sepra) columns and gel filtration on Superdex 200 prep grade (GE Healthcare) where Complex I is eluted as a single peak with the expected 550 kDa molecular mass (Euro *et al*., 2009).

### Spheroplast preparation

The *E. coli* cells were converted to spheroplasts by treatment with 0.1 mg ml^−1^ lysozyme in the presence of 30% sucrose.

### Measurements of catalytic activity

HAR and DQ reductase activities of purified or membrane-bound Complex I were measured by following (d)NADH oxidation at 340 nm (ε = 6.2 mM^−1^·cm^−1^) in the basic buffer containing 25 mM HEPES-BTP [1,3-bis(tris(hydroxymethyl)methylamino)propane], pH 7.5, and 3.5 mM KCl. Concentrations of added substrates were 80 µM for DQ, 360 µM for HAR and 100 µM for (d)NADH. For the measurements of the ubiquinone reductase activity of purified solubilized Complex I, the basic buffer was supplemented with 0.005% DDM (n-dodecyl β-d-maltopyranoside) and 20 nM *bo_3_* ubiquinol oxidase. For measurements of the catalytic activity in spheroplasts the basic buffer was supplemented with 0.4 M mannitol to prevent spheroplast breakage, and 18 µg ml^−1^ alamethicin to allow dNADH penetration into the cytoplasm.

### Analytical procedures

Protein concentrations in membranes was determined by the BCA protein assay reagent kit and for purified protein Pierce 660 nm protein assay reagent was used (both from Thermo Scientific). Bovine serum albumin was used as a standard in both cases.

### Proton pumping activity measurements

Inverted membrane vesicles for the measurements of ΔpH generation were prepared as described in Euro *et al*. (2008), but the concentration of buffer loaded into the vesicles, 100 mM HEPES-KOH, pH 7.5, was increased by a factor of two. Energy-dependent acidification of the interior of membrane vesicles was followed by the fluorescence change of the pH-sensitive probe, acridine orange, AO (λ_ex_ = 493 nm, λ_em_ = 530 nm). The medium contained 5 µM AO, 200 mM HEPES-KOH, pH 7.5, 50 mM K_2_SO_4_, 0.8 mM MgSO_4_, 0.1 µM valinomycin and 5 mM KCN and 100 µM DQ or 30 µM Q1 when indicated. The membrane protein concentration was 50 µg ml^−1^ in all tests. The reaction was initiated by the addition of 80 µM dNADH or 100 µM DQ as indicated; gramicidin at 1 µg ml^−1^ was added to dissipate generated ΔpH. The fluorescence changes were measured with a Hitachi F-7000 fluorescence spectrophotometer. All measurements of the activity or proton pumping were reproduced two to four times using independent membrane preparations for each mutant.

## References

[b1] Belevich G, Euro L, Wikström M, Verkhovskaya M (2007). Role of the conserved arginine 274 and histidine 224 and 228 residues in the NuoCD subunit of Complex I from *Escherichia coli*. Biochemistry.

[b2] Bryson K, McGuffin LJ, Marsden RL, Ward JJ, Sodhi JS, Jones DT (2005). Protein structure prediction servers at University College London. Nucleic Acids Res.

[b3] Efremov RG, Sazanov LA (2011). Structure of the membrane domain of respiratory complex I. Nature.

[b4] Efremov RG, Baradaran R, Sazanov LA (2010). The architecture of respiratory complex I. Nature.

[b5] Euro L, Belevich G, Verkhovsky MI, Wikstrom M, Verkhovskaya M (2008). Conserved lysine residues of the membrane subunit NuoM are involved in energy conversion by the proton-pumping NADH:ubiquinone oxidoreductase (Complex I). Biochim Biophys Acta.

[b6] Euro L, Belevich G, Wikstrom M, Verkhovskaya M (2009). High affinity cation-binding sites in Complex I from *Escherichia coli*. Biochim Biophys Acta.

[b7] Fearnley IM, Walker JE (1992). Conservation of sequences of subunits of mitochondrial complex-I and their relationships with other proteins. Biochim Biophys Acta.

[b9] Galkin A, Drose S, Brandt U (2006). The proton pumping stoichiometry of purified mitochondrial complex I reconstituted into proteoliposomes. Biochim Biophys Acta.

[b8] Galkin AS, Grivennikova VG, Vinogradov AD (1999). –>H+/2e- stoichiometry in NADH-quinone reductase reactions catalyzed by bovine heart. FEBS Lett.

[b10] Green GN, Kranz RG, Lorence RM, Gennis RB (1984). Identification of subunit-I as the cytochrome-b558 component of the cytochrome-d terminal oxidase complex of *Escherichia coli*. J Biol Chem.

[b11] Hiramatsu T, Kodama K, Kuroda T, Mizushima T, Tsuchiya T (1998). A putative multisubunit Na^+^/H^+^ antiporter from *Staphylococcus aureus*. J Bacteriol.

[b12] Hunte C, Zickermann V, Brandt U (2010). Functional modules and structural basis of conformational coupling in mitochondrial complex I. Science.

[b1001] Link AJ, Phillips D, Church GM (1997). Methods for generating precise deletions and insertions in the genome of wild-type *Escherichia coli*: application to open reading frame characterization. J Bacteriol.

[b13] Mathiesen C, Hagerhall C (2003). The ‘antiporter module’ of respiratory chain Complex I includes the MrpC/NuoK subunit – a revision of the modular evolution scheme. FEBS Lett.

[b14] Morino M, Natsui S, Ono T, Swartz TH, Krulwich TA, Ito M (2010). Single site mutations in the hetero-oligomeric Mrp antiporter from alkaliphilic *Bacillus pseudofirmus* OF4 that affect Na^+^/H^+^ antiport activity, sodium exclusion, individual Mrp protein levels, or Mrp complex formation. J Biol Chem.

[b15] Nakamaru-Ogiso E, Kao MC, Chen H, Sinha SC, Yagi T, Ohnishi T (2010). The membrane subunit NuoL(ND5) is involved in the indirect proton pumping mechanism of *Escherichia coli* complex I. J Biol Chem.

[b17] Ohnishi T (2010). STRUCTURAL BIOLOGY Piston drives a proton pump. Nature.

[b16] Ohnishi T, Nakamaru-Ogiso E (2008). Were there any ‘misassignments’ among iron-sulfur clusters N4, N5 and N6b in NADH-quinone oxidoreductase (complex I)?. Biochim Biophys Acta.

[b18] Puustinen A, Finel M, Virkki M, Wikström M (1989). Cytochrome *o**bo*) is a proton pump in *Paracoccus denitrificans* and *Escherichia coli*. FEBS Lett.

[b19] Puustinen A, Finel M, Haltia T, Gennis RB, Wikström M (1991). Properties of the two terminal oxidases of *Escherichia coli*. Biochemistry.

[b21] Sazanov LA (2007). Respiratory complex I: mechanistic and structural insights provided by the crystal structure of the hydrophilic domain. Biochemistry.

[b20] Sazanov LA, Carroll J, Holt P, Toime L, Fearnley IM (2003). A role for native lipids in the stabilization and two-dimensional crystallization of the *Escherichia coli* NADH-ubiquinone oxidoreductase (complex I). J Biol Chem.

[b22] Steimle S, Bajzath C, Dörner K, Schulte M, Bothe V, Friedrich T (2011). Role of subunit NuoL for proton translocation by respiratory complex I. Biochemistry.

[b24] Verkhovskaya M, Knuuti J, Wikström M (2011). Role of Ca^2+^ in structure and function of Complex I from *Escherichia coli*. Biochim Biophys Acta.

[b23] Verkhovskaya ML, Belevich N, Euro L, Wikström M, Verkhovsky MI (2008). Real-time electron transfer in respiratory complex I. Proc Natl Acad Sci USA.

[b25] Wikström M (1984). Two protons are pumped from the mitochondrial matrix per electron transferred between NADH and ubiquinone. FEBS Lett.

[b26] Zickermann V, Drose S, Tocilescu MA, Zwicker K, Kerscher S, Brandt U (2008). Challenges in elucidating structure and mechanism of proton pumping NADH:ubiquinone oxidoreductase (complex I). J Bioenerg Biomembr.

