# Supplementary material for: Probing the mechanistic role of the long α-helix in subunit L of respiratory Complex I from Escherichia coli by site-directed mutagenesis
Source: Mol Microbiol. 2011 Nov 7;82(5):1086–95. doi: 10.1111/j.1365-2958.2011.07883.x (PMC3274701; doi:10.1111/j.1365-2958.2011.07883.x)

Supplementary data for:

Probing the mechanistic role of the long α-helix in subunit L of respiratory Complex I from *Escherichia coli* by site-directed mutagenesis

Galina Belevich, Juho Knuuti, Michael I. Verkhovsky, Mårten Wikström, Marina Verkhovskaya

Helsinki Bioenergetics Group, Institute of Biotechnology, PO Box 65 (Viikinkaari 1) FIN-00014 University of Helsinki, Finland

Table S1
Oligonucleotide primers used for cloning and mutagenesis of *E.coli nuoL* gene

Primer	Sequence [a]
for PCR	
UpLF	ATGGCAGGTATGGCAATCGACG
UpLXhoR	GGTTAA**CT**C**G**AGCATGTTCATCCG
DnLXhoF	GC**T**C**GA**GGGCATATAAGAACGTAG
DnLR	ACAGCGGCAGGGAGAAACG
UpLNheR	GGTTAAGGC**T**AGCATGTTCATCCG
LnNheF	ATTCCGCT**A**G**C**TTATCTCTGCTTCC
for mutagenesis [b]	
Y590St	TGCTGTTAAGTGAGAACGGCTA**A**CTGCGCTGGTAT
D542N	GGTGGTACAACGCCTGGGGATTT**A**ACTGGCTGTATG
D542R	GGTACAACGCCTGGGGATTT**CG**CTGGCTGTATGACAA
K551Q	ATGACAAAGTGTTCGTC**C**AGCCGTTCCTGGGTATT
K551E	ATGACAAAGTGTTCGTC**G**AGCCGTTCCTGGGTATT
P552A	CAAAGTGTTCGTCAAG**G**CGTTCCTGGGTATTGC
P552Q	CAAAGTGTTCGTCAAGC**A**GTTCCTGGGTATTGCCT
P552C	TATGACAAAGTGTTCGTCAAG**TGC**TTCCTGGGTATTGCCTGGTTG
G532ins[6]	GCCGTCTGCTGGGCGATGGTGATGGTGACCCTACCTGGTGGTACAAC
G532ins[7]	CCGTCTGCTGGGCCCAGATGGTGATGGTGACCCTACCTGGTGGTACAAC
G532sub[7]	CAACAGTGCGCCGGGCCGTCTGCTGGGC**C**C**AGATG**G**CG**AC**GG**CG**ATCC**GGGATTT GACTGGCTGTATGACAAAGTG
P552ins[6]	GTGTTCGTCAAGCCGGATGGTGATGGTGACCCATTCCTGGGTATTGCC
P552ins[7]	GTGTTCGTCAAGCCGCCAGATGGTGATGGTGACCCTTTCCTGGGTATTGCC
P564ins[6]	TGCTGAAACGCGATCCGGATGGTGATGGTGACCCACTGAACTCAATGATGAAC
P564sub[7]	CCTGGGTATTGCCTGGTTGCTGAAACGCGATCCG**GGTG**AC**GG**T**GATGGTGA**C**CCA** CCGGCTGTCCTTTCCCGCTTTGCAGGTAAAGGTC
P572ins[6]	CAATGATGAACATCCCGGATGGTGATGGTGACCCAGCTGTCCTTTCCCGCT

[a] New restriction sites are underlined, altered nucleotides are in bold, and nucleotide sequence in insertions/substitutions is shaded
[b] Sequence represents sense primer, antisense primer had reverse complementary sequence

Fig.S1. Growth curves of NuoL mutants truncated at N- and C-termini and with single amino acid substitution in AH in minimal malate medium.

GR70N, wild type, black squares; GRL3 (ΔnuoL), red diamonds; GRLn, cyan stars; Y590St, blue, solid line and squares; K551fs, blue, dashed line, open cycles; D542N, green, solid line and squares; D542R, green, dashed line, open squares; K551Q, dark yellow, solid lines and triangles; K551E, dark yellow, dashed line, open triangles; P552A, wine, solid lines and cycles; P552C, wine, dashed line, open cycles; P552Q, wine, dotted line, open cycles with cross.

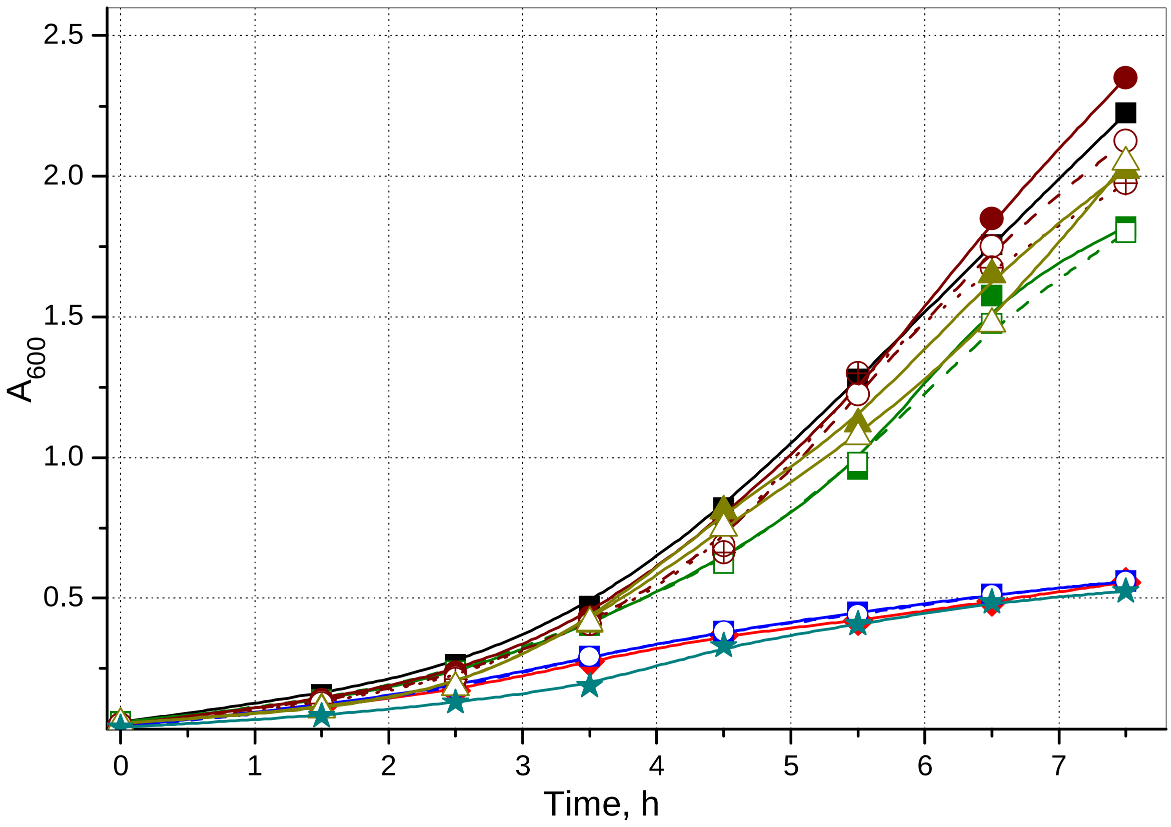

Fig.S2. Growth curves of NuoL mutants with insertion/substitution in AH in minimal malate medium.

GR70N, wild type, black squares; GRL3 (ΔnuoL), red diamonds; G542*ins*^{7}, wine, solid line and cycles; G542*ins*^{6}, wine, dashed line, open cycles; G542*sub*^{7}, wine, dotted line, open cycles with cross, P552*ins*^{6}, green, solid line and triangles; P552*ins*^{7}, green, dashed lane, open triangles; P564*ins*^{6}, blue, solid line and diamonds; P654*sub*^{7}, blue, dashed line, open diamonds; P572*ins*^{6}, dark yellow stars.

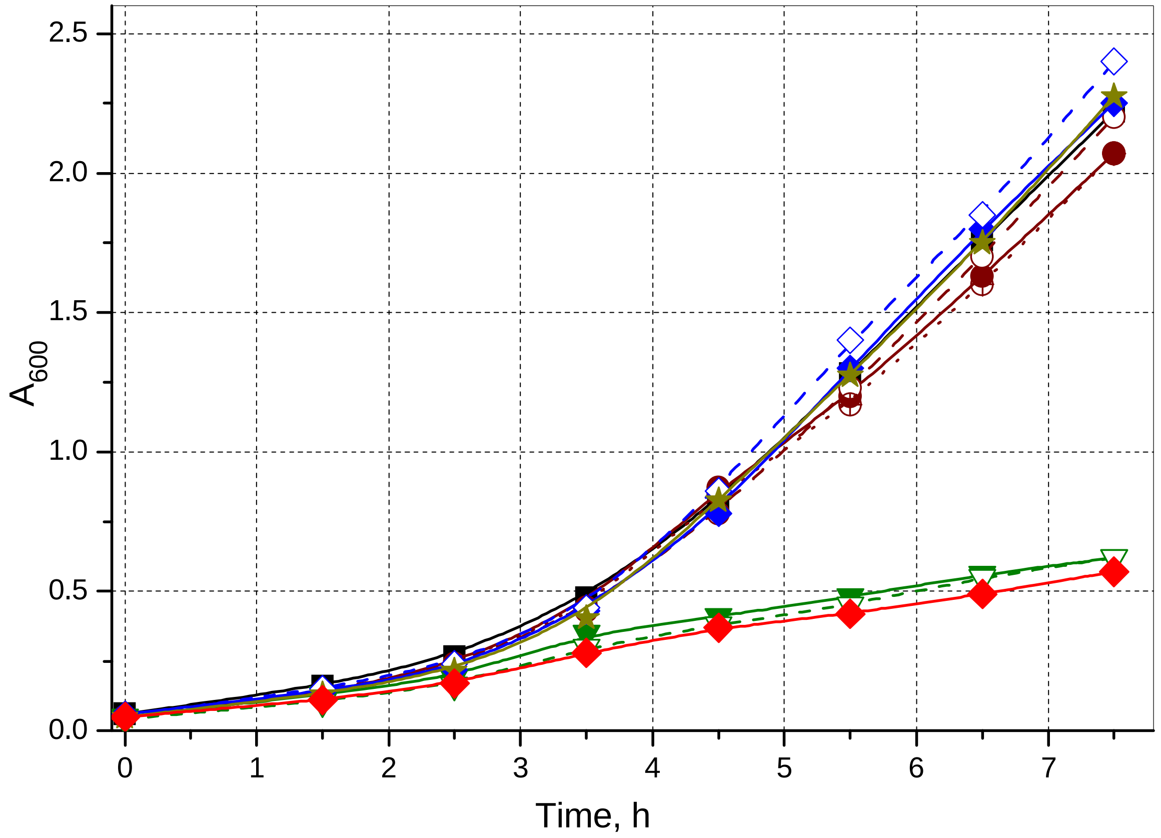

Fig.S3. SDS-PAGE of purified *E. coli* Complex I.

Lane 1, molecular mass markers (kDa). Lane 2, P552ins[6] variant. Lane 3, wild type. The assignment of the observed bands to the individual subunits of Complex I is given on the right.

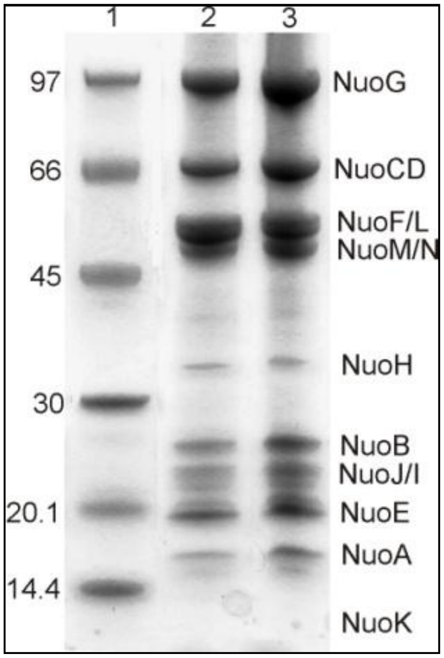

Supplement: Supplementary file 1 [file mmi0082-1086-SD1.pdf]
